# Combined percutaneous and transurethral cystoscopy for large urethral foreign bodies: A case report and literature review

**DOI:** 10.1016/j.eucr.2021.101625

**Published:** 2021-03-04

**Authors:** Ahmed Albakr, Khalid Al Rumaihi, Abdulla Al Ansari, Abdulkader Alobaidy

**Affiliations:** Department of Urology, Hamad Medical Corporation, Doha, Qatar

**Keywords:** Foreign body, Urethra, Percutaneous cystoscopy

## Abstract

Since early reports in the literature, cases with urethral foreign bodies have been extremely variable and interestingly challenging. Various presentations have been reported, including self-insertion for sexual pleasure, assaults, trauma, and foreign bodies migration. Management by endoscopic surgery is usually a preferred option for small foreign bodies. Open surgery is reserved for large objects with difficult access. We present a case of self-insertion of a large urethral foreign body with a caliber of 45F in a young gentleman. Endoscopic extraction of the foreign body was safely successful using combined percutaneous and transurethral cystoscopy.

## Introduction

Numerous foreign body insertion incidents have been reported since early literature. Foreign bodies inserted included rubber, screws, bullets, wires, surgical instruments up to dead animals like snakes and fish.^1^ Incidents ranged from self-insertion, iatrogenic, assault, and migrating foreign bodies. The best answer for the management question should represent the characters of minimal invasiveness, having fewer long-term complications, and ensuring early recovery.

We present a case of self-insertion of a large foreign body in the urethra, a large width of the foreign body (45 French) rubber rod. We also demonstrate the successful minimally invasive technique for the removal of such a large object.

## Case presentation

A 39-year-old healthy gentleman presented to the emergency department 6 hours after he self-inserted a silicon rod into his urethra. He was unable to pass urine and complaining of lower abdominal pain. The patient claimed that he was trying to relieve a feeling of urethral irritation and dysuria. After insertion, he noticed some blood at the urethral meatus and later developed retention of urine. He was unable to retrieve the foreign body. The patient denied having similar episodes before and had no previous correlated medical or surgical history.

**Fig. 1:**
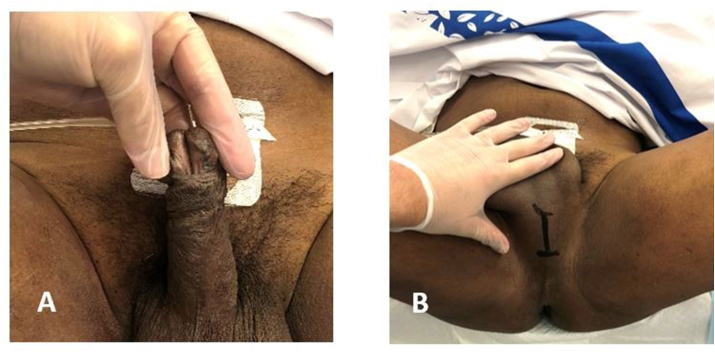
(A) Distal penile hypospadias found on examination. (B) Location of the foreign body by palpation of the perineal area.

Abdominal examination revealed distended bladder with suprapubic bulge and tenderness. With genital examination, we noticed that the patient had a circumcised penis with distal penile hypospadias (Fig. 1-A). We could palpate an approximately 8 cm foreign material in the bulbar urethra (Fig. 1-B). The distal end could not be felt or milked towards the urethral meatus. A suprapubic catheter (SPC) 12 French (F) was inserted under ultrasound guidance to relieve the patient urinary retention draining clear urine. X-ray failed to visualize the exact location of the foreign body as it was radiolucent. A decision was made to prepare the patient for cystoscopy for a trial of removal of the foreign body.

## Intraoperative findings

**Fig. 2:**
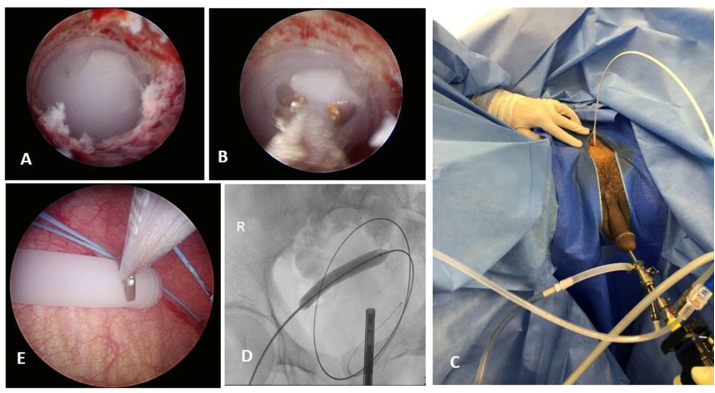
(A–B) Foreign body entrapped in the bulbar urethra failed to be grasped by ureteroscope forceps. (C–D) Balloon dilatation of the suprapubic access. (E) Grasping the foreign body from the bladder by the nephroscope forceps through the percutaneous tract.

Under spinal anesthesia and after skin preparation, urethroscopy using cystoscope 21 F showed multiple erosions in the anterior urethra with one distal urethral perforation. Silicon foreign body was noticed with its distal end fitting into the bulbar urethral lumen (Fig. 2-A, B). Trials to grasp the foreign body with cystoscope forceps failed because it was slippery. Ureteroscope 9.5 French failed to find any other passages around the object and its forceps failed to grasp it. The foreign body was pushed to the bladder using the ureteroscope under vision with difficulty. After relieving the obstruction, the cystoscope showed a high bladder neck and a foreign body with an estimated length of 7 cm free-floating in the bladder.

The foreign body was removed using percutaneous cystoscopy. A super-stiff guide wire 0.035 was inserted into the bladder through the SPC under cystoscopy. Dilatation of the suprapubic track was done with 8F then 10 F coaxial dilators. A Nephromax balloon was used to dilate the track inflated with contrast material under fluoroscopy guidance. Alken rod was inserted over the guidewire under vision. Further dilatation was done using Amplatz dilator (28 F) followed by introducing the working sheath (30 F). Nephroscope (26 F) was advanced through the working sheath. The foreign body was conical, with one end broader than the other. The broader end was difficult to pass through the working sheath. Finally, the foreign body was grasped with difficulty using nephroscope double action jaws forceps from the narrower end (Fig. 2- C, D, E).

**Fig. 3:**
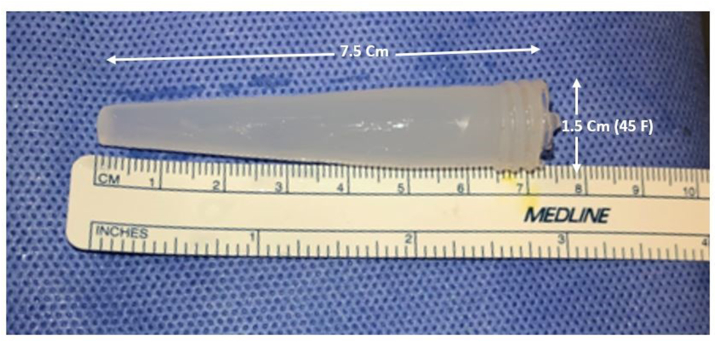
The silicon rod after removal with a length of 7.5 cm and a diameter at the widest part of 1.5 cm.

While pulling it through the sheath, the foreign material distal half was stuck in the sheath, so the sheath was pulled with the foreign material. The object (Fig. 3) was delivered slowly through the skin. A percutaneous track 1 cm in width was sutured using a single stitch of an absorbable suture. A urethral catheter 16 F was inserted over a guidewire. The patient had a smooth postoperative course and was discharged the next day with a urethral catheter. After five days, the catheter was removed in the clinic, and the patient could void smoothly. The patient did not attend his follow-up appointments and refused to do a urethrogram after catheter removal.

## Discussion

Removal of foreign bodies from the male urethra tends to be challenging and difficult, especially with large objects. The foreign body removed was conical and had a length of 7.5 cm. The wider end caliber was 1.5 cm (45 F width). The object width is larger than the average urethral meatus width reported in uncircumcised adults to be 22–26F and less in circumcised males.^2^ The introduction of such an object was only possible as the patient had hypospadias.

The use of percutaneous cystoscopy has been described before for the management of urinary bladder foreign bodies. Wegner et al., in 1997, reported the successful removal of 2 pencils from the bladder of a 21-year-old female through percutaneous nephrolithotomy sheath and forceps.^3^ Johnin et al., in 1999, reported the use of percutaneous nephrolithotomy sheath and forceps for removal of 11 sewing needles on a fishing line that was inserted into the urethra of a 38-year-old gentleman. The needles were pushed into the urethra using a Nelaton catheter.^4^ Interestingly this case reported also had a history of hypospadias and previous foreign body insertion attempts into the urethra. To our knowledge, the case we are reporting is the first case that used combined transurethral and percutaneous cystoscopy for removal of a large and wide caliber foreign body.

## Conclusion

Although removing large foreign bodies from the male urethra tends to be difficult, the use of combined percutaneous and transurethral cystoscopy has been effective, less invasive, and associated with early recovery.

## Declaration of competing interest

The authors have no conflict of interest to declare.
